# *Michelia compressa*-Derived Santamarine Inhibits Oral Cancer Cell Proliferation via Oxidative Stress-Mediated Apoptosis and DNA Damage

**DOI:** 10.3390/ph17020230

**Published:** 2024-02-09

**Authors:** Hsin-I Lu, Kuan-Liang Chen, Ching-Yu Yen, Chung-Yi Chen, Tsu-Ming Chien, Chih-Wen Shu, Yu-Hsuan Chen, Jiiang-Huei Jeng, Bing-Hung Chen, Hsueh-Wei Chang

**Affiliations:** 1 Graduate Institute of Medicine, College of Medicine, Kaohsiung Medical University, Kaohsiung 80708, Taiwan; u112500009@gap.kmu.edu.tw; 2 Department of Dentistry, Chi-Mei Medical Center, Tainan 71004, Taiwan; 870712@mail.chimei.org.tw (K.-L.C.); ycy@tmu.edu.tw (C.-Y.Y.); 3 School of Dentistry, Taipei Medical University, Taipei 11031, Taiwan; 4 Department of Nutrition and Health Sciences, School of Medical and Health Sciences, Fooyin University, Kaohsiung 83102, Taiwan; xx377@fy.edu.tw; 5 Department of Urology, Kaohsiung Medical University Hospital, Kaohsiung 80756, Taiwan; 1020431@kmuh.edu.tw; 6 Department of Urology, Faculty of Medicine, College of Medicine, Kaohsiung Medical University, Kaohsiung 80708, Taiwan; 7 Institute of BioPharmaceutical Sciences, National Sun Yat-sen University, Kaohsiung 80424, Taiwan; cwshu@g-mail.nsysu.edu.tw; 8 Department of Biomedical Science and Environmental Biology, Bachelor Program of Life Sciences, College of Life Science, Kaohsiung Medical University, Kaohsiung 80708, Taiwan; u110024003@gap.kmu.edu.tw; 9 School of Dentistry, College of Dental Medicine, Kaohsiung Medical University, Kaohsiung 80708, Taiwan; jhjeng@kmu.edu.tw; 10 Department of Dentistry, Kaohsiung Medical University Hospital, Kaohsiung 80708, Taiwan; 11 Department of Dentistry, National Taiwan University Hospital, Taipei 100225, Taiwan; 12 Department of Biotechnology, Kaohsiung Medical University, Kaohsiung 80708, Taiwan; 13 Center for Cancer Research, Kaohsiung Medical University, Kaohsiung 80708, Taiwan; 14 Department of Medical Research, Kaohsiung Medical University Hospital, Kaohsiung Medical University, Kaohsiung 80708, Taiwan

**Keywords:** evergreen tree, natural product, oxidative stress, apoptosis, oral cancer

## Abstract

The anti-oral cancer effects of santamarine (SAMA), a *Michelia compressa* var. compressa-derived natural product, remain unclear. This study investigates the anticancer effects and acting mechanism of SAMA against oral cancer (OC-2 and HSC-3) in parallel with normal (Smulow–Glickman; S-G) cells. SAMA selectively inhibits oral cancer cell viability more than normal cells, reverted by the oxidative stress remover *N*-acetylcysteine (NAC). The evidence of oxidative stress generation, such as the induction of reactive oxygen species (ROS) and mitochondrial superoxide and the depletion of mitochondrial membrane potential and glutathione, further supports this ROS-dependent selective antiproliferation. SAMA arrests oral cancer cells at the G2/M phase. SAMA triggers apoptosis (annexin V) in oral cancer cells and activates caspases 3, 8, and 9. SAMA enhances two types of DNA damage in oral cancer cells, such as γH2AX and 8-hydroxy-2-deoxyguanosine. Moreover, all of these anticancer mechanisms of SAMA are more highly expressed in oral cancer cells than in normal cells in concentration and time course experiments. These above changes are attenuated by NAC, suggesting that SAMA exerts mechanisms of selective antiproliferation that depend on oxidative stress while maintaining minimal cytotoxicity to normal cells.

## 1. Introduction

Oral cancer is among the top 10 most-occurring cancers and is prevalent globally [1]. There are several primary factors that cause oral cancer, such as drinking, smoking, and betel nut chewing [2]. The most common oral cancer cell type is oral squamous cell carcinoma (OSCC), at higher than 90% [3]. Although radio- or chemo-therapy [4] can improve oral cancer treatment, their benefits are halted by the accompanied adverse effects [5,6]. Hence, it is essential to identify additional drugs with fewer side effects in oral cancer therapy.

The *Michelia* genus belongs to the Magnoliaceae family, containing around 30 species. One of these species, *Michelia compressa* var. compressa, is an evergreen tree predominantly found in the Ryukyu Islands, Japan, and Taiwan. Indigenous populations use this *Michelia* species as a traditional herb for cancer treatments. For instance, *M. hypoleuca* and *M. officinalis* have been employed in China to treat cancerous sores and leukemia [7]. *M. alba* shows antimicrobial, anti-inflammatory, and antidiabetic effects [8]. *M. champaca* has been utilized to address abdominal tumors in India and shows antidepressant effects for neuropsychiatric disorders [9].

Many sesquiterpene lactones exhibit anticancer properties [10] and may show antioxidant [11,12] or pro-oxidant functions [13], depending on their structures, concentrations, and cell types. Antioxidants at high concentrations potentially induce oxidative stress that improves the antiproliferation of cancer cells [14,15]. Therefore, this oxidative stress-modulating potential of sesquiterpene lactones may contribute to their anticancer effects.

Santamarine (SAMA), a sesquiterpene lactone derivative C_15_H_20_O_3_ [16], can be isolated from *M. lanuginosa*, *Centaurea uniflora*, *Eupatorium capillifolium* [17], *Inula helenium*, and *I. japonica* [18]. SAMA exhibits antioxidant effects against H_2_O_2_ in human dermal fibroblasts [19]. Hence, the anticancer effects of SAMA warrant an evaluation. Recently, several antiproliferative effects of SAMA have been reported on cervical, liver, and lung cancer [18,20,21,22]. For example, SAMA inhibits thioredoxin reductase (TrxR), contributing to its anticancer effect in cervical cancer cells [20]. This inhibition of TrxR shows oxidative stress-inducing effects and causes apoptosis, as reported in other TrxR inhibitors such as sanguinarine. Similarly, SAMA induces oxidative stress to trigger apoptosis of liver [21] and lung [22] cancer cells. 

Low cytotoxicity to normal cells can alleviate the potential side effects of anticancer drugs. However, the anticancer properties of SAMA for oral cancer have rarely been investigated. The safety of the drug SAMA has not been thoroughly assessed in examining its cytotoxicity to normal cells.

This study examines the proliferation-modulating response between oral cancer cells and normal cells to assess SAMA’s potential selective antiproliferation function. Moreover, the mechanism of the selective antiproliferation of SAMA was uncovered in parallel between oral cancer cells and normal cells by evaluating their cell viability, cell cycle disruption, oxidative stress, and DNA damage response. This study thereby offers a detailed assessment of the underlying mechanism against oral cancer.

## 2. Results

### 2.1. Proliferation-Regulating Effects of SAMA: Oral Cancer Cells vs. Normal Cells

**Figure 1:**
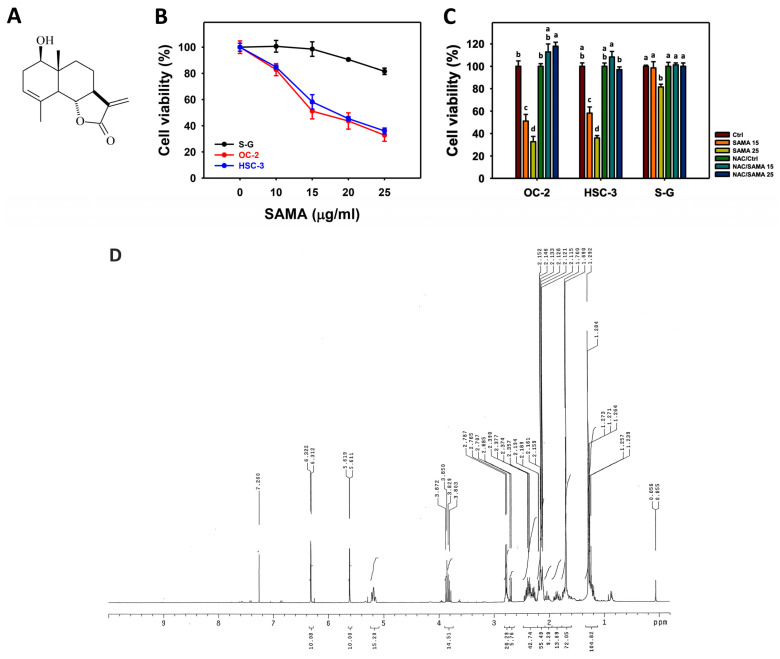
SAMA shows antiproliferation of oral cancer cells. Viability was evaluated using the CCK-8 method with 24 h drug treatments. (**A**) SAMA structure. (**B**) Cell viability of SAMA. Oral cancer (OC-2 and HSC-3) and normal (S-G) cell lines were treated with 0.1% DMSO (untreated control) and SAMA for 24 h. (**C**) Cell viability of NAC/SAMA. NAC/SAMA represents the pre-treatment of 10 mM NAC for 1 h and the post-treatment of SAMA (0, 15, and 25 μg/mL) for 24 h. The SAMA (15 and 25 μg/mL) treatments are divided into SAMA 15 and SAMA 25. (**D**) ^1^H NMR spectrum of SAMA. An absence of overlapping annotations (lowercase notes) between treatments indicated a significant difference (*p* < 0.05). Data are shown as means ± SD (triplicate). For the statistical example in Figure 1C (OC-2 cells), the SAMA 0 (ctrl), 15, and 25 μg/mL treatments labeled with “b, c, and d” represent significant differences.

SAMA (Figure 1A) shows dose–response inhibition of oral cancer cell viability (OC-2 and HSC-3) (Figure 1B), indicating IC_50_ values of 15.7 and 18.49 μg/mL, respectively. The normal cell viability (Smulow–Glickman; S-G) is weakly influenced by SAMA. This suggests that SAMA has selective antiproliferation properties for oral cancer cells.

Since oxidative stress is an essential factor for antiproliferation, its involvement in SAMA treatment was assessed using *N*-acetylcysteine (NAC). The differential antiproliferation by SAMA between oral cancer cells and normal cells was suppressed by NAC (NAC/SAMA) (Figure 1C). The ^1^H NMR spectrum of SAMA is shown in Figure 1D.

### 2.2. Cell Cycle-Modulating Effects of SAMA: Oral Cancer Cells vs. Normal Cells

**Figure 2:**
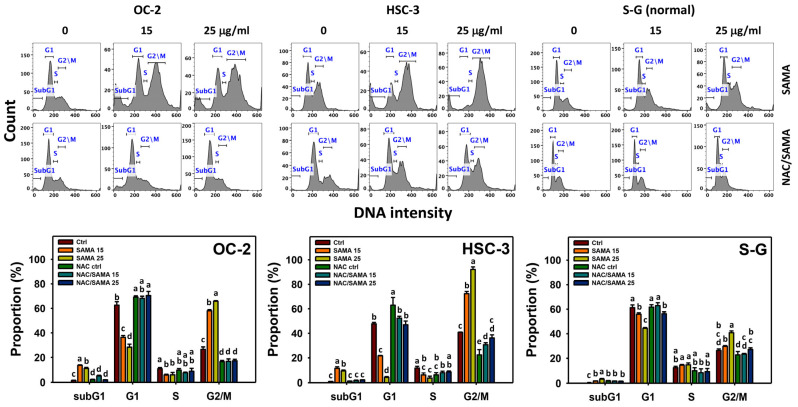
SAMA shows cell cycle disturbance of oral cancer cells. NAC/SAMA represents the pre-treatment of 10 mM NAC for 1 h and the post-treatment of SAMA (0, 15, and 25 μg/mL) for 24 h. The SAMA (15 and 25 μg/mL) treatments are divided into SAMA 15 and SAMA 25. Data are shown as means ± SD (triplicate). An absence of overlapping annotations (lowercase notes) between treatments indicated a significant difference (*p* < 0.05).

SAMA marginally elevates the subG1 phase in oral cancer (OC-2 and HSC-3) compared to normal (S-G) cells. SAMA decreases the G1 and S phases and increases the G2/M phase in oral cancer cells (Figure 2), indicating that SAMA causes G2/M arrest. By comparison, the G1, S, and G2/M phases of normal oral cells are only minorly changed by SAMA.

Moreover, the involvement of SAMA treatment in cell cycle progression was evaluated using NAC. The differential cell cycle disturbance by SAMA between oral cancer cells and normal cells was suppressed by NAC (NAC/SAMA) (Figure 2).

### 2.3. Apoptosis-Modulating Effects of SAMA: Oral Cancer Cells vs. Normal Cells

**Figure 3:**
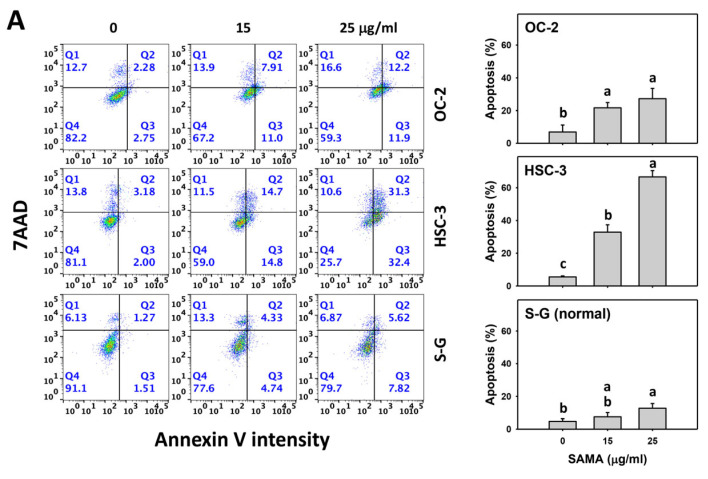

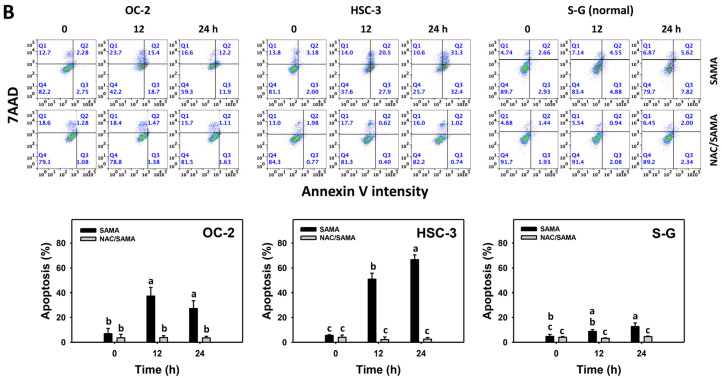
SAMA shows apoptosis in oral cancer cells. (**A**) Annexin V/7-aminoactinmycin D (7AAD) assay of SAMA. Cells were treated with 0.1% DMSO (untreated control) and SAMA (0, 15, and 25 μg/mL) for 24 h. The proportion of annexin V (+)/7AAD (±) (%) was regarded as apoptosis (+) (%). (**B**) Annexin V/7AAD assay of NAC/SAMA. NAC/SAMA represents the pre-treatment of 10 mM NAC for 1 h and the post-treatment of 25 μg/mL SAMA for 0, 12, and 24 h. Data are shown as means ± SD (triplicate). An absence of overlapping annotations (lowercase notes) between treatments indicated a significant difference (*p* < 0.05).

The annexin V assay is a regular tool for evaluating apoptotic cells showing phosphatidylserine in the outer plasma membrane [23]. SAMA increases the annexin V (+) (%) of oral cancer cells in a dose–response manner (Figure 3A), while it shows a weak change in normal (S-G) cells. 

Moreover, the involvement of SAMA treatment in apoptosis was assessed using NAC. SAMA increases the annexin V (+) (%) of oral cancer cells at each time interval (Figure 3B), while it shows a weak change in normal cells. The differential apoptosis induction by SAMA between oral cancer cells and normal cells was suppressed by NAC (NAC/SAMA).

### 2.4. Caspase 3-Modulating Effects of SAMA: Oral Cancer Cells vs. Normal Cells

**Figure 4:**
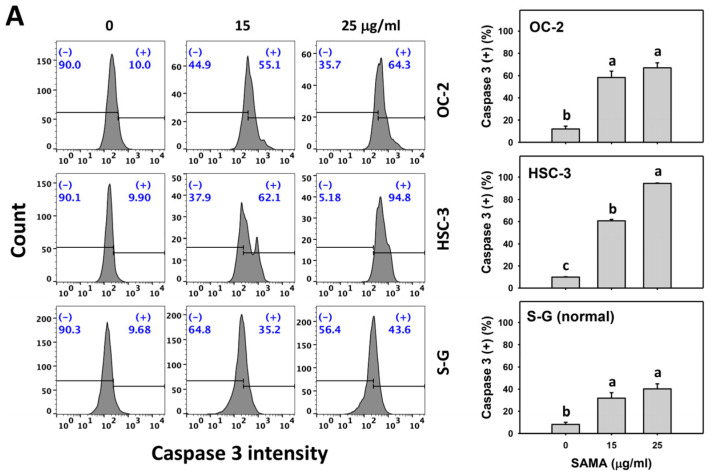

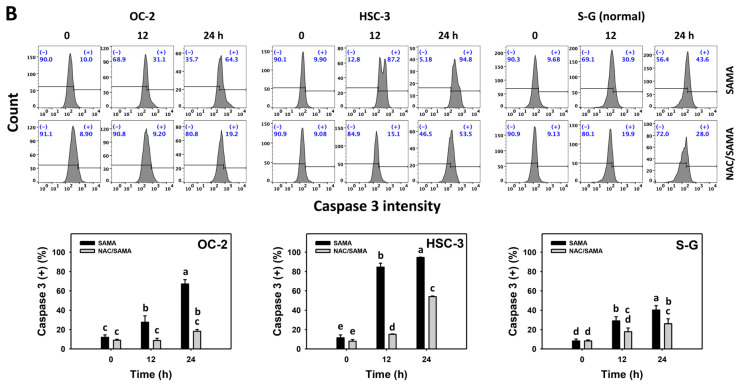
SAMA shows caspase 3 activation in oral cancer cells. (**A**) Caspase 3 activation assays of SAMA. Cells were treated with 0.1% DMSO (untreated control) and SAMA (0, 15, and 25 μg/mL) for 24 h. (+) is assigned to the caspase 3 (+) (%) region. (**B**) Caspase 3 activation assay of NAC/SAMA. NAC/SAMA represents the pre-treatment of 10 mM NAC for 1 h and the post-treatment of 25 μg/mL SAMA for 0, 12, and 24 h. Data are shown as means ± SD (triplicate). An absence of overlapping annotations (lowercase notes) between treatments indicated a significant difference (*p* < 0.05).

Caspase 3 activation is a central biomarker for apoptotic signaling [24], which is detectable with flow cytometry [25,26]. The caspase 3 (+) (%) is proportional to caspase 3 activation. When the concentrations increase, SAMA increases the caspase 3 (+) (%) in oral cancer cells more than in S-G cells (Figure 4A).

Moreover, the involvement of SAMA treatment in caspase 3 activation was assessed using NAC. SAMA increases caspase 3 (+) (%) of oral cancer cells at each time interval (Figure 4B), while it shows a minor change in normal cells. The differential activation of caspase 3 by SAMA between oral cancer cells and normal cells was suppressed by NAC (NAC/SAMA).

### 2.5. Caspase 8- and 9-Modulating Effects of SAMA: Oral Cancer Cells vs. Normal Cells

**Figure 5:**
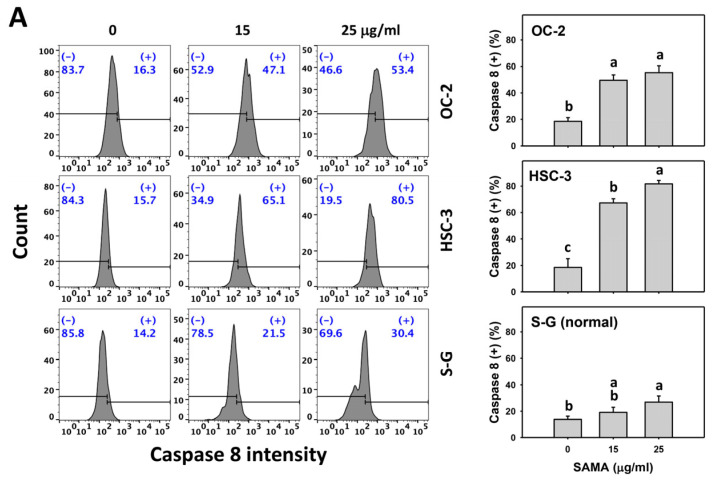

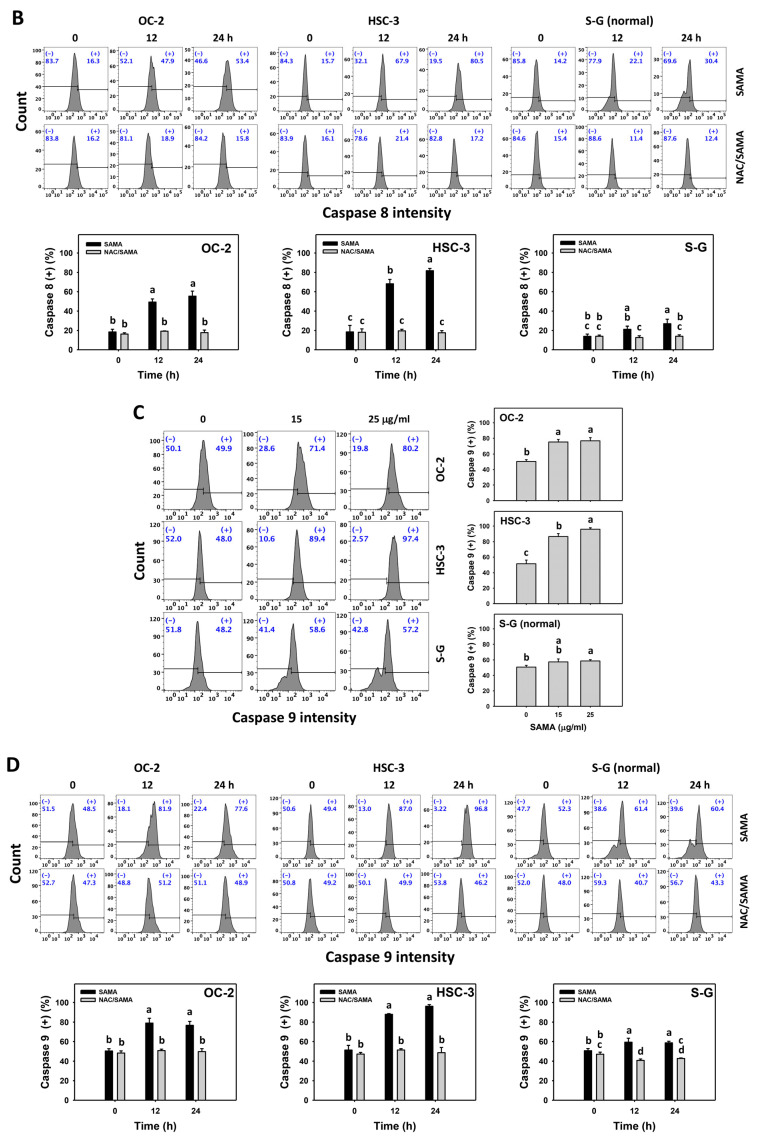
SAMA activates caspases 8 and 9 in oral cancer cells. (**A**,**C**) Caspase 8 and 9 activation assays of SAMA. Cells were treated with 0.1% DMSO (untreated control) and SAMA (0, 15, and 25 μg/mL) for 24 h. (+) is assigned to the caspase 8 and 9 (+) (%) region. (**B**,**D**) Caspase 8 and 9 assays with NAC/SAMA. NAC/SAMA represents the pre-treatment of 10 mM NAC for 1 h and the post-treatment of 25 μg/mL SAMA for 0, 12, and 24 h. Data are shown as means ± SD (triplicate). An absence of overlapping annotations (lowercase notes) between treatments indicated a significant difference (*p* < 0.05).

Caspases 8 and 9 and their activation are biomarkers for extrinsic and intrinsic apoptotic signaling [24], which are detectable with flow cytometry [25,26]. The caspases 8 and 9 (+) (%) are proportional to caspase 8 and 9 activation. When the concentrations increase, SAMA increases the caspases 8 and 9 (+) (%) in oral cancer cells more than in S-G cells (Figure 5A,C).

Moreover, the involvement of SAMA treatment in the activation of caspases 8 and 9 was assessed using NAC. SAMA increases caspases 8 and 9 (+) (%) in oral cancer cells at each time interval (Figure 5B,D), while it shows a weak change in normal cells. The differential activation of caspases 8 and 9 by SAMA between oral cancer cells and normal cells was suppressed by NAC (NAC/SAMA).

### 2.6. Oxidative Stress (Reactive Oxygen Species (ROS) and Mitochondrial Superoxide (MitoSOX))-Modulating Effects of SAMA: Oral Cancer Cells vs. Normal Cells

**Figure 6:**
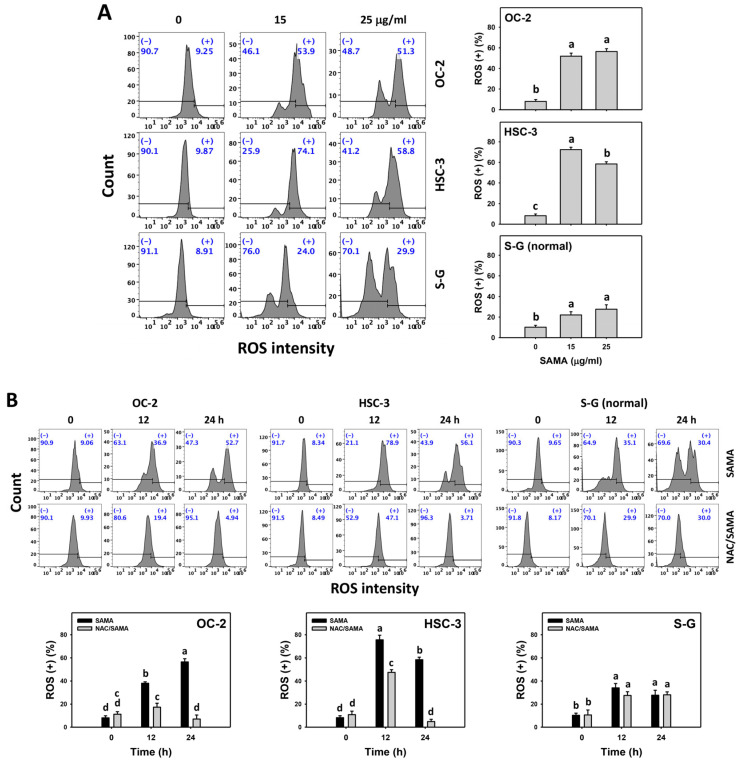
SAMA shows ROS upregulation in oral cancer cells. (**A**) ROS assay for SAMA. Cells were treated with 0.1% DMSO (untreated control) and SAMA (0, 15, and 25 μg/mL) for 24 h. (+) is assigned to the ROS (+) (%) region. (**B**) ROS assay for NAC/SAMA. NAC/SAMA represents the pre-treatment of 10 mM NAC for 1 h and the post-treatment of 25 μg/mL SAMA for 0, 12, and 24 h. Data are shown as means ± SD (triplicate). An absence of overlapping annotations (lowercase notes) revealed a significant difference (*p* < 0.05).

**Figure 7:**
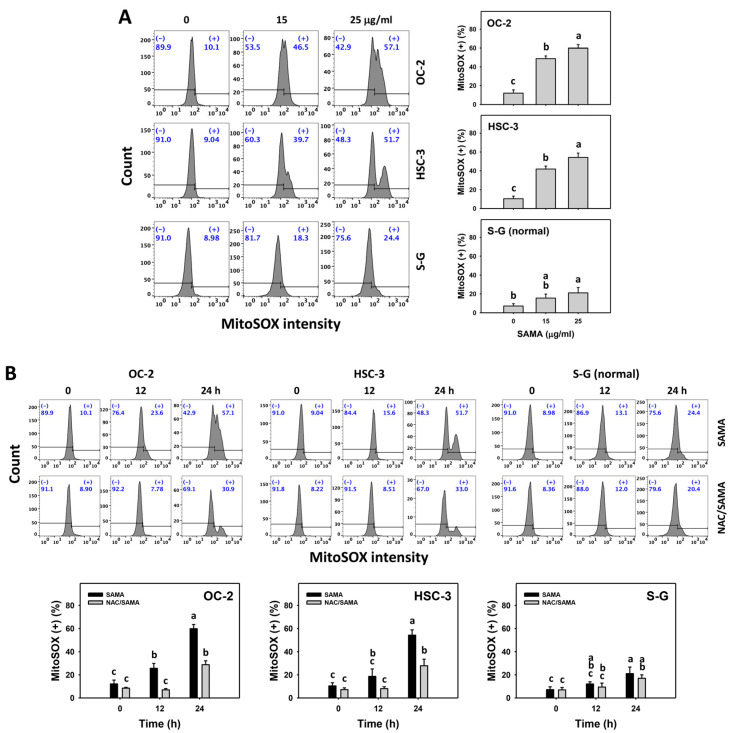
SAMA shows MitoSOX upregulation in oral cancer cells. (**A**) MitoSOX assay for SAMA. Cells were treated with 0.1% DMSO (untreated control) and SAMA (0, 15, and 25 μg/mL) for 24 h. (+) is assigned to the MitoSOX (+) (%) region. (**B**) MitoSOX assay for NAC/SAMA. NAC/SAMA represents the pre-treatment of 10 mM NAC for 1 h and the post-treatment of 25 μg/mL SAMA for 0, 12, and 24 h. Data are shown as means ± SD (triplicate). An absence of overlapping annotations (lowercase notes) between treatments indicated a significant difference (*p* < 0.05).

As mentioned above, NAC validates the role of oxidative stress in several SAMA-associated changes (Figure 1, Figure 2, Figure 3, Figure 4 and Figure 5). However, direct evidence of oxidative stress is absent and this warrants further assessment, such as by employing ROS and MitoSOX flow cytometry [27]. The oral cancer cells at different concentrations of SAMA treatments exhibited more highly increased ROS and MitoSOX (+) (%) than the S-G cells (Figure 6A and Figure 7A).

Moreover, the involvement of SAMA treatment in ROS and MitoSOX generation was assessed using NAC. SAMA increases the ROS and MitoSOX (+) (%) of oral cancer cells at each time interval (Figure 6B and Figure 7B), while it shows a weak change in normal cells. The differential generation of oxidative stress by SAMA between oral cancer cells and normal cells was suppressed by NAC (NAC/SAMA).

### 2.7. Mitochondrial Membrane Potential (MMP)-Modulating Effects of SAMA: Oral Cancer Cells vs. Normal Cells

**Figure 8:**
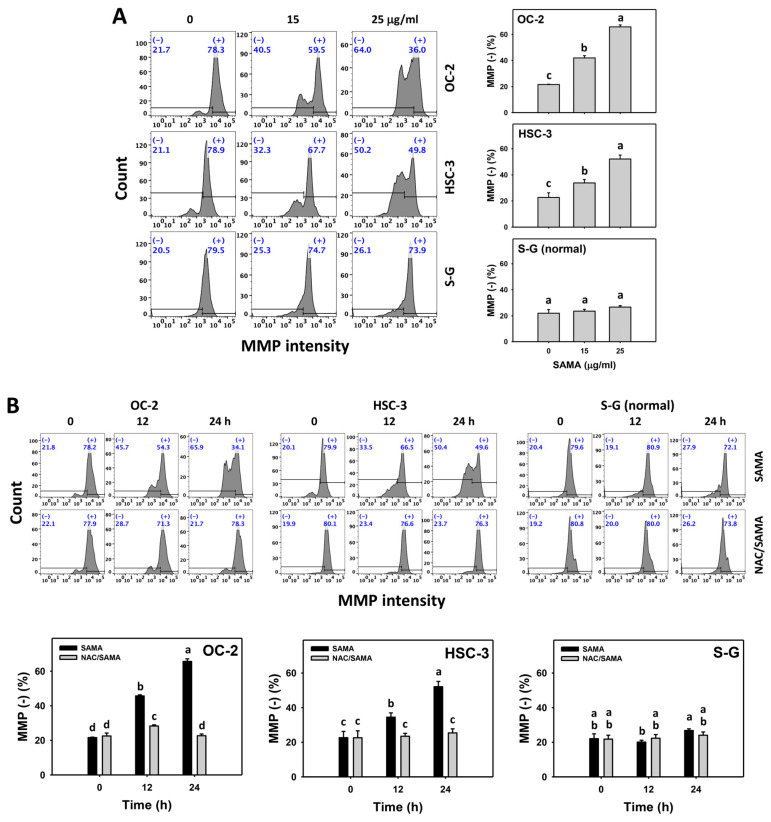
SAMA shows MMP downregulation in oral cancer cells. (**A**) MMP assay for SAMA. Cells were treated with 0.1% DMSO (untreated control) and SAMA (0, 15, and 25 μg/mL) for 24 h. (−) is assigned to the MMP (−) (%) region. (**B**) MMP assay for NAC/SAMA. NAC/SAMA represents the pre-treatment of 10 mM NAC for 1 h and the post-treatment of 25 μg/mL SAMA for 0, 12, and 24 h. Data are shown as means ± SD (triplicate). An absence of overlapping annotations (lowercase notes) between treatments indicated a significant difference (*p* < 0.05).

Under oxidative stress, MMP depletion occurs [20,21], which is detected with flow cytometry [28]. The MMP (−) (+) (%) is proportional to MMP depletion. SAMA dose-responsively increases the MMP (−) (+) (%) in oral cancer cells more than in S-G cells (Figure 8A).

Moreover, the involvement of SAMA treatment in MMP depletion was assessed using NAC. SAMA time-dependently increases the MMP (−) (+) (%) of oral cancer cells (Figure 8B), while it shows a weak change in normal S-G cells. The differential depletion of MMP (−) by SAMA between oral cancer cells and normal S-G cells was suppressed by NAC (NAC/SAMA).

### 2.8. Glutathione (GSH)-Modulating Effects of SAMA: Oral Cancer Cells vs. Normal Cells

**Figure 9:**
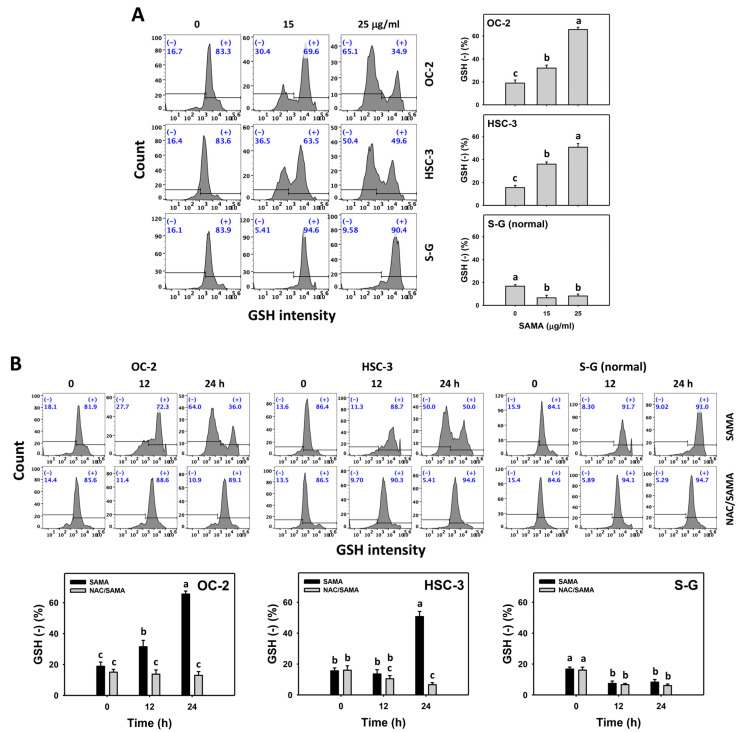
SAMA shows GSH downregulation in oral cancer cells. (**A**) GSH assay for SAMA. Cells were treated with 0.1% DMSO (untreated control) and SAMA (0, 15, and 25 μg/mL) for 24 h. (−) is assigned to the GSH (−) (%) region. (**B**) GSH assay for NAC/SAMA. NAC/SAMA represents the pre-treatment of 10 mM NAC for 1 h and the post-treatment of 25 μg/mL SAMA for 0, 12, and 24 h. Data are shown as means ± SD (triplicate). An absence of overlapping annotations (lowercase notes) between treatments indicated a significant difference (*p* < 0.05).

GSH downregulation can enhance oxidative stress [14], which can be detected with flow cytometry [28]. The GSH (−) (+) (%) is proportional to GSH depletion. SAMA dose-responsively increases the GSH (−) (+) (%) in oral cancer cells more than in S-G cells (Figure 9A).

Moreover, the involvement of SAMA treatment in GSH depletion was assessed using NAC. SAMA increases the GSH (−) (+) (%) of oral cancer cells over time (Figure 9B), while it shows a weak change in normal cells. The differential depletion of MMP (−) by SAMA between oral cancer cells and normal cells was suppressed by NAC (NAC/SAMA).

### 2.9. DNA Damage-Modulating Effects of SAMA: Oral Cancer Cells vs. Normal Cells 

**Figure 10:**
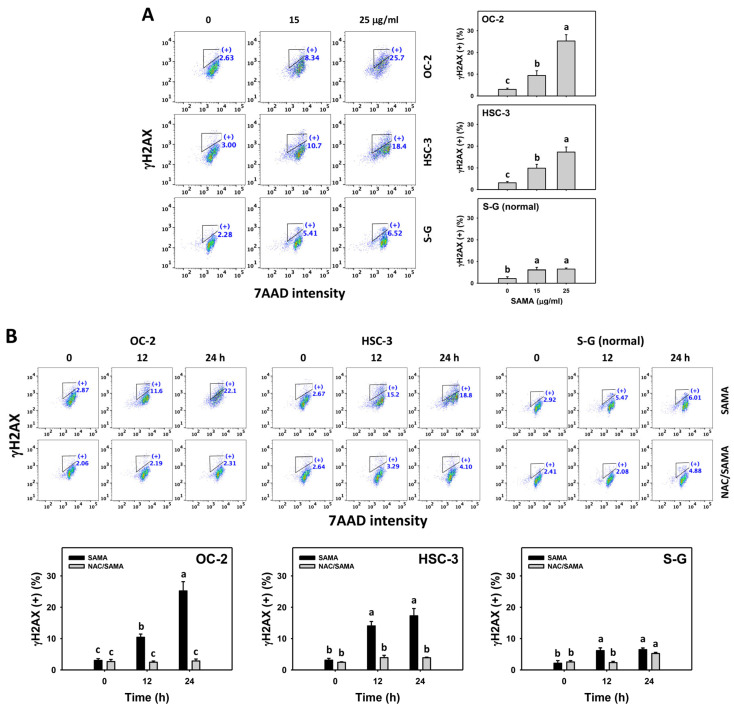
SAMA shows γH2AX upregulation in oral cancer cells. (**A**) γH2AX assay for SAMA. Cells were treated with 0.1% DMSO (untreated control) and SAMA (0, 15, and 25 μg/mL) for 24 h. (+) is assigned to the γH2AX (+) (%) region. (**B**) γH2AX assay for NAC/SAMA. NAC/SAMA represents the pre-treatment of 10 mM NAC for 1 h and the post-treatment of 25 μg/mL SAMA for 0, 12, and 24 h. Data are shown as means ± SD (triplicate). An absence of overlapping annotations (lowercase notes) between treatments indicated a significant difference (*p* < 0.05).

**Figure 11:**
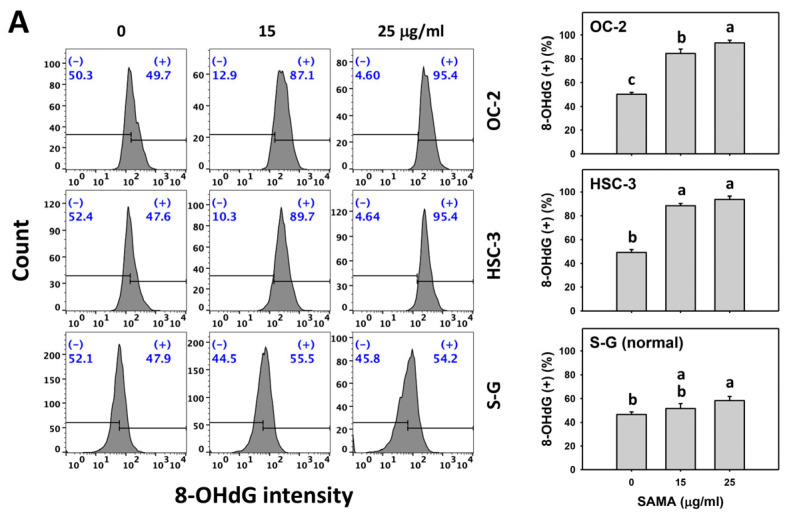

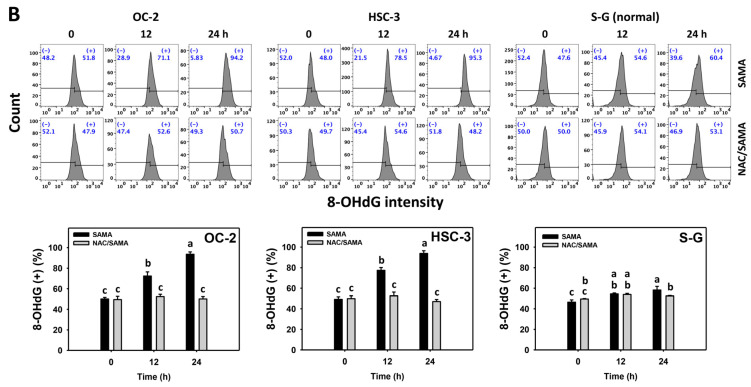
SAMA shows 8-OHdG upregulation in oral cancer cells. (**A**) 8-OHdG assay for SAMA. Cells were treated with control (0.1% DMSO) and SAMA (0, 15, and 25 μg/mL) for 24 h. (+) is assigned to the 8-OHdG (+) (%) region. (**B**) 8-OHdG assay for NAC/SAMA. NAC/SAMA represents the pre-treatment of 10 mM NAC for 1 h and the post-treatment of 25 μg/mL SAMA for 0, 12, and 24 h. Data are shown as means ± SD (triplicate). An absence of overlapping annotations (lowercase notes) between treatments indicated a significant difference (*p* < 0.05).

Under oxidative stress, DNA damage occurs [14], which is detectable with flow cytometry, such as γH2AX and 8-hydroxy-2-deoxyguanosine (8-OHdG) [28]. The γH2AX and 8-OHdG (+) (%) are proportional to their DNA damage levels. SAMA dose-responsively increases the γH2AX and 8-OHdG (+) (%) in oral cancer cells more than in S-G cells (Figure 10A and Figure 11A).

Moreover, the involvement of SAMA treatment in γH2AX and 8-OHdG generation was assessed using NAC. SAMA time-dependently increases the γH2AX and 8-OHdG (+) (%) of oral cancer cells (Figure 10B and Figure 11B), while it shows a weak change in normal cells. The differential depletion of MMP (−) by SAMA between oral cancer cells and normal cells was suppressed by NAC (NAC/SAMA).

## 3. Discussion

The antiproliferative effects of SAMA and their acting mechanisms were addressed in this anti-oral cancer study. The treatment safety of SAMA was also assessed by analyzing it with normal cells. Several anti-oral cancer mechanisms were discussed as follows.

### 3.1. Antiproliferative Effects of SAMA in Different Types of Cancer Cells

SAMA shows anticancer effects in cervical [20], liver [21], and lung [22] cancer cells, i.e., the IC_50_ values of SAMA in a 24 h MTT assay are 40, 70, and ~60 μM for HeLa, HepG2, and A549, respectively. By comparison, SAMA in a 24 h CCK-8 assay showed IC_50_ values of 15.7 and 18.49 μg/mL (63.3 and 74.5 μM) in oral cancer cells (OC-2 and HSC-3), respectively. Although different viability assays have been performed, the antiproliferative effects of SAMA are common in several cancer cell types, showing broad-spectrum antitumor effects. By comparison, cisplatin exhibits a higher sensitivity to oral cancer (Ca9-22) cells in a 24 h CCK-8 assay, i.e., its IC_50_ value is 5.6 μg/mL [28]. However, cisplatin has the potential side effects of hepatotoxicity and nephrotoxicity [29,30].

In contrast, SAMA shows the same low cytotoxicity to normal liver [21] and lung [21,22] cells compared to their cancer cells. Similarly, SAMA showed low toxicity to normal oral cells (S-G), i.e., an 80% viability at the highest test concentration (25 μg/mL). Therefore, SAMA has the potential effect of inducing more antiproliferation against oral cancer cells than normal cells. This suggests that SAMA has moderate drug safety in normal cells and may exhibit low adverse effects when applied in oral cancer treatment.

### 3.2. SAMA Causes Oxidative Stress of Oral Cancer Cells

Drug-induced overload of cellular oxidative stress is an effective strategy for cancer treatment [14,28,31,32]. The ROS induction may be tolerated by normal cells but overloads cancer cells [14], leading to the selective antiproliferation of cancer cells. Several drugs show selective ROS generation and antiproliferation in cancer cells. For instance, methanol extracted from *Theonella swinhoei* (METS) [33] and fucoidan [28] exhibit higher oxidative stress and antiproliferation in oral cancer cells than in normal cells. Consistently, SAMA inhibits proliferation by upregulating oral cancer cell oxidative stress, as evidenced by the production of ROS and MitoSOX and the depletion of MMP (Figure 6, Figure 7 and Figure 8).

The source of oxidative stress is generally derived from the imbalance of redox homeostasis [14], such as the downregulation of cellular antioxidants. Several drugs, such as METS [33] and fucoidan [28], downregulate the GSH, causing oxidative stress for antiproliferation in oral cancer cells [34]. Consistently, SAMA depletes the GSH of oral (Figure 9), liver [21], and lung [22] cancer cells, accompanied by upregulating oxidative stress. This finding supports that SAMA downregulates GSH to upregulate oxidative stress. 

Moreover, SAMA suppresses antioxidant signaling, such as TrxR, contributing to oxidative stress induction in cervical [20], liver [21], and lung [22] cancer cells. The thioredoxin (Trx) system, consisting of nicotinamide adenine dinucleotide phosphate (NADPH), TrxR, and Trx, has an important antioxidant function in attenuating oxidative stress [35]. Accordingly, more antioxidant signaling pathways need to be investigated to explore the oxidative stress responses in SAMA treatment of oral cancer cells in the future.

### 3.3. SAMA Causes G2/M Arrest, Apoptosis, and DNA Damage

Oxidative stress is a multi-functional effector for inducing G2/M arrest [36,37], apoptosis [38], and DNA damage [14]. For example, tert-butyl hydroperoxide stimulates oxidative stress to cause G2/M arrest in liver cancer cells [36]. Several anticancer drugs exhibit G2/M arrest, leading to apoptosis later. 2-amino 9-chloro-7-(2-fluorophenyl)-5*H*-pyrimidol[5,4-d][2]benzazepine promotes G2/M arrest, and in turn, this triggers apoptosis of prostate and breast cancer cells [39]. Sinularin promotes ROS generation in order to induce G2/M arrest and apoptosis in liver [37] cancer cells. Fucoidan causes apoptosis and DNA damage in oral cancer cells via increasing ROS [28]. 3,4,5-trimethoxy-4’-bromo-cis-stilbene initially triggers G2/M arrest and subsequently promotes apoptosis of lung cancer cells [40]. Benzyl isothiocyanate causes DNA damage to pancreatic cancer cells, leading to G2/M arrest and apoptosis [41].

Consistently, SAMA stimulates oxidative stress and induces G2/M arrest of oral cancer cells, accompanied by a minor subG1 increase (Figure 2). Cells may be led to apoptosis without subG1 accumulation because it is not an absolute apoptotic indicator [42]. In certain instances, no evident subG1 proportions are observed in drug-triggered apoptosis, which varies depending on the duration of drug exposure. For example, *Aaptos suberitoides* extract induces apoptosis and caspase activation without evident subG1 accumulation in breast cancer cells [43]. The effects of subG1 proportions of 24 and 48 h treatments of (−)-anonaine on lung cancer cells are few, but they dramatically increase at 72 h [44]. Consequently, the validation of drug-induced apoptosis needs further assessment through other assays, as is described later in the paper.

In addition to G2/M arrest, SAMA also induces oxidative stress to promote an increase in annexin V intensity (Figure 3) and upregulates the activation of apoptosis signaling (caspase 3) (Figure 4). Either intrinsic (caspase 9) or extrinsic (caspase 8) upstream signaling is upregulated in oral cancer cells (Figure 5). Similarly, SAMA activates caspases 3, 8, and 9 in liver cancer cells [21]. Furthermore, SAMA promotes oxidative stress to upregulate oral cancer cell γH2AX and 8-OHdG, the DNA damage markers (Figure 10 and Figure 11).

### 3.4. Selective Antiproliferation Mechanism of SAMA

An ideal cancer treatment must selectively inhibit the proliferation of cancer cells while leaving normal cells unharmed. The current study validates that SAMA inhibits more proliferation of oral cancer cells than normal cells, indicating that SAMA exhibits a selective antiproliferation function with drug safety. After assessment, the oxidative stress induction (ROS, MitoSOX, MMP, and GSH) (Figure 6, Figure 7, Figure 8 and Figure 9) is higher in oral cancer cells than in normal cells, suggesting the selective production of oxidative stress in oral cancer cells. This induces several mechanisms, such as G2/M arrest, apoptosis, the activation of caspases 3, 8, and 9, and DNA damage occurrence (γH2AX and 8-OHdG) to a greater extent in oral cancer cells than in normal cells (Figure 2, Figure 4, Figure 5, Figure 10 and Figure 11). The participation of oxidative stress in these mechanisms was validated by the NAC, which reverts the mechanism of selective antiproliferation. Consequently, SAMA exhibits ROS-dependent selective antiproliferation mechanisms in oral cancer cells.

When compared its effects in cervical [20], liver [21], lung [22], and oral cancer cells, as in the present study, SAMA consistently enhances oxidative stress and apoptosis. Liver [21] and lung [22] cancer cells show that SAMA inhibits the nuclear factor kappa-light-chain-enhancer of B cell (NF-κB) activation. Moreover, liver cancer cells further demonstrate that SAMA also suppresses the signal transducer and activator of transcription 3 (STAT3) activation [22]. In comparison, we found that SAMA promotes G2/M arrest, MitoSOX, and DNA damage (γH2AX and 8-OHdG) in oral cancer cells, which have not been investigated in other cancer cells. According to these different cancer studies, the potential anticancer strategy of SAMA involves the modulation of GSH and antioxidant signaling (TrxR) to enhance cell cycle arrest at G2/M, cellular and mitochondrial oxidative stress, and DNA damage, leading to apoptosis of cancer cells. Notably, this proposed anticancer strategy of SAMA in oral cancer cells still needs further validation for some of these responses.

### 3.5. Potential Application of SAMA in Combined Treatment

Many natural products exhibit synergistic anticancer effects when combined with clinical drugs [45,46,47,48], but drug toxicity to normal cells may limit the application of such combined treatments in cancer therapy. The goal of combination therapy would be to sensitize the tumor to existing treatments and possibly allow lower doses and fewer side effects. SAMA exhibits a broad-spectrum antitumor function, as described here (Section 3.1), with drug safety to the normal liver [21], lung [21], and oral cells (Figure 1B), suggesting that SAMA may have the potential to be used for monotherapy and combined treatment to oral and other cancer cells.

The neurokinin-1 receptor (NK-1R) is generally overexpressed in several cancer cells compared to benign lesions [49], e.g., oral cancer [50]. Several combined treatment studies involving NK-1R antagonists and anticancer drugs have been reported [49,51], particularly for the application of drug resistance [52]. Conventional therapies, such as cisplatin [47], epidermal growth factor receptor (EGFR) inhibitors [53,54], and radiation [55], are also commonly used for combined treatment with natural products. Consequently, combination therapy using SAMA and NK-1R antagonists, cisplatin, EGFR inhibitors, and radiation may synergistically improve oral cancer treatment with low side effects towards normal cells.

### 3.6. Future Research of SAMA in Oral Cancer Treatment

As described, SAMA induces oxidative stress in oral cancer cells, causing apoptosis. Moreover, oxidative stress leads to non-apoptosis types of cancer cell death, such as autophagy and ferroptosis [56,57]. A future thorough examination of non-apoptotic oral cell death in SAMA treatment is necessary.

## 4. Materials and Methods

### 4.1. Preparation of SAMA

*M. compressa* var. compressa fruits were gathered from Chiayi County, Taiwan, and soaked in methanol to isolate the SAMA. To elaborate, 3.6 kg of air-dried fruits were extracted with MeOH (5 L × 3) at room temperature. After concentration by reducing the pressure, 46.2 g MeOH extract was collected and chromatographed over silica gel (810 g, 70–230 mesh), using a mixture of *n*-hexane/EtOAc/MeOH as eluents. Then, it yielded four fractions. From fraction 1, 7.2 g was used to perform further silica gel chromatography, eluted with a gradually enriched *n*-hexane-EtOAc (60:1) mixture, producing five fractions (1-1–1-5). From fractions 2–3, 1.4 g was isolated on a silica gel column using the *n*-hexane/EtOAc mixtures, resulting in the preparation of SAMA (5 mg) [7]. The ^1^H-NMR spectrum was measured on a Varian Unity Inova spectrometer at 400 MHz (Agilent Technologies, Santa Clara, CA, USA).

### 4.2. Oxidative Stress Remover

The involvement of oxidative stress in the SAMA experiments was evaluated by pre-treating the cells with 10 mM NAC (Sigma-Aldrich, St. Louis, MO, USA) for 1 h before SAMA treatment [58,59].

### 4.3. Cell Culture and Viability Assay

The gingival epithelial cell line S-G was used for the normal cells [60,61,62]. Two oral cancer cell lines were applied in this study, namely HSC-3 (JCR Bank, Ibaraki, Osaka, Japan) and OC-2 [63] (kindly given by Kaohsiung Medical University (Dr. Wan-Chi Tsai)). The cultural maintenance and medium preparation have been described previously [64]. Cell viability was evaluated with a CCK-8 reagent (IMT Formosa New Materials, Kaohsiung, Taiwan) [65] for 1 h.

### 4.4. Cell Cycle

A 75% ethanol solution was applied for cell fixation. Following overnight cooling, cells were washed with PBS. Then, 1 μg/mL 7AAD (Biotium, Inc., Hayward, CA, USA) was added to the cell suspension for 30 min [66]. Finally, cells were washed and resuspended with PBS for Guava easyCyte flow cytometry (Luminex, Austin, TX, USA). This 7AAD-stained DNA content was utilized to determine the phases of the cell cycle.

### 4.5. Apoptosis

Following the manufacturer’s instructions, the cells underwent 30 min staining with annexin V/7AAD reagent (1:1000/1 μg/mL) provided by Strong Biotech Inc., Taipei, Taiwan. After PBS washing, the occurrence of apoptosis was assessed with flow cytometry (Luminex, Austin, TX, USA) [28,67].

### 4.6. Caspase 3, 8, and 9 Activation

The activation statuses of caspases 3, 8, and 9 were identified using flow cytometry kits (OncoImmunin, Gaithersburg, MD, USA) [25,26] by incubating at 37 °C for 1 h (1:1000). The caspase 3-, 8-, and 9-specific peptides were provided by commercial kits, i.e., PhiPhiLux-G1D2, CaspaLux8-L1D2, and CaspaLux9-M1D2. Upon activation, the respective peptide substrates of caspases 3, 8, and 9 underwent cleavage, forming fluorescent peptides. These peptides, originating from caspase-activated cells, were then assessed by flow cytometry (Guava easyCyte, Luminex, Austin, TX, USA).

### 4.7. ROS and MitoSOX Content

Oxidative stress-reacting dyes, specifically 2′,7′-dichlorodihydrofluorescein diacetate (DCFH-DA) [28,68] (Molecular Probes, Invitrogen, Eugene, OR, USA) and MitoSOX™ Red [69] (Sigma-Aldrich, St. Louis, MO, USA), were selected for ROS and MitoSOX detection. The reacting conditions for DCFH-DA and MitoSOX™ Red were 2 and 5 μM for 30 min, respectively. Following this, the amount of ROS and MitoSOX in the stained cells was assessed with flow cytometry.

### 4.8. MMP Content

MMP depletion is one of the indicators of oxidative stress [28]. An MMP-reacting dye, specifically DiOC_2_(3) (Invitrogen, San Diego, CA, USA), was selected for MMP detection. DiOC2(3)’s reacting conditions were 50 nM for 20 min. Following this, the amount of MMP in the stained cells was assessed with flow cytometry.

### 4.9. GSH Content

GSH depletion is one of the indicators of oxidative stress [34]. A GSH-reacting dye, specifically 5-chloromethylfluorescein diacetate (CMF-DA) (Thermo Fisher Scientific, Carlsbad, CA, USA), was selected for GSH detection. The reacting condition for CMF-DA was 5 μM for 20 min. Following this, the amount of GSH in the stained cells was assessed using flow cytometry.

### 4.10. γH2AX DNA Damage

γH2AX is an early biomarker of DNA double-strand breaks [28]. A 75% ethanol solution was applied for cell fixation. Following overnight cooling, the cells were washed with PBS. Then, the γH2AX antibody (Santa Cruz Biotechnology, Santa Cruz, CA, USA) was introduced (1:500). Then, ethanol-fixed cells were mixed with the Alexa Fluor^®^488 secondary antibody (Cell Signaling Technology, Beverly, MA, USA) and DNA dye (7AAD) for 30 min. Following this, the amount of γH2AX in the drug-treated cells was assessed via flow cytometry.

### 4.11. 8-OHdG DNA Damage

8-OHdG is a common form of oxidative damage to DNA [28]. A 75% ethanol solution was applied for cell fixation. Following overnight cooling, the cells were washed with PBS. Then, fixed cells were mixed with an FITC-labeled 8-OHdG antibody (Santa Cruz Biotechnology) for 30 min. Following this, the amount of 8-OHdG in the drug-treated cells was assessed with flow cytometry.

### 4.12. Statistical Analysis

The JMP12 software (SAS Institute, Cary, NC, USA) was employed to conduct an ANOVA with Tukey’s HSD test to ascertain the significance of multiple comparisons. JMP12 provides lowercase notes that are assigned to the column of each treatment. An absence of overlapping annotations (lowercase notes) between the treatments indicated a significant difference (*p* < 0.05). Data were derived from three separate experiments, presented as the mean ± standard deviation (SD).

## 5. Conclusions

The suppressing effects of proliferation on oral cancer cells by the *M. compressa* var. compressa derivative SAMA has rarely been explored. In this study, the selective antiproliferation properties of SAMA were validated in oral cancer cells, leaving normal cells only slightly affected. The selective impact of the antiproliferation of SAMA is mediated by oxidative stress, as corroborated by testing with NAC. The evidence of oxidative stress generation induced by SAMA was validated in higher levels in the oral cancer cells than in the normal cells. Both cellular ROS and MitoSOX were predominantly upregulated, and MMP and GSH were downregulated in the oral cancer cells. Concerning other non-cancer studies of SAMA, the potential role of TrxR and other signal transductions, such as NF-κB and STAT3, in suppressing oxidative stress warrants an advanced assessment in oral cancer treatment.

Furthermore, SAMA treatment for oral cancer cells enhances apoptosis and activates caspases 3, 8, and 9, as reported in cervical, liver, and lung cancer cells. Although no detailed signaling was assessed, this study provides a novel finding that SAMA inflicts more G2/M arrest and DNA damage, such as γH2AX and 8-OHdG, in oral cancer cells than in normal cells. All of these selective mechanisms for antiproliferation exerted by SAMA are attenuated by NAC. In addition to apoptosis, a thoughtful investigation of non-apoptosis cell death of oral cancer in SAMA treatment still needs to be conducted in the future. In conclusion, this study demonstrates that SAMA exhibits oxidative stress-mediated antiproliferation properties against oral cancer with low cytotoxicity to normal cells.

## Data Availability

Data is contained within the article.

## References

[1] Rivera C. (2015). Essentials of oral cancer. Int. J. Clin. Exp. Pathol..

[2] Chung C.H., Yang Y.H., Wang T.Y., Shieh T.Y., Warnakulasuriya S. (2005). Oral precancerous disorders associated with areca quid chewing, smoking, and alcohol drinking in southern Taiwan. J. Oral Pathol. Med..

[3] Almangush A., Makitie A.A., Triantafyllou A., de Bree R., Strojan P., Rinaldo A., Hernandez-Prera J.C., Suarez C., Kowalski L.P., Ferlito A. (2020). Staging and grading of oral squamous cell carcinoma: An update. Oral Oncol..

[4] Liao Y.H., Chou W.Y., Chang C.W., Lin M.C., Wang C.P., Lou P.J., Chen T.C. (2023). Chemoprevention of oral cancer: A review and future perspectives. Head Neck.

[5] Riva G., Cravero E., Pizzo C., Briguglio M., Iorio G.C., Cavallin C., Ostellino O., Airoldi M., Ricardi U., Pecorari G. (2022). Sinonasal side effects of chemotherapy and/or radiation therapy for head and neck cancer: A literature review. Cancers.

[6] Silverman S. (1999). Oral cancer: Complications of therapy. Oral Surg. Oral Med. Oral Pathol. Oral Radiol. Endod..

[7] Wang S.J., Wang T.H., Kao C.L., Yeh H.C., Li H.T., Chen C.Y. (2023). Secondary metabolites of fruits of *Michelia compressa* var. compressa. Chem. Nat. Compd..

[8] Cheng K.K., Nadri M.H., Othman N.Z., Rashid S., Lim Y.C., Leong H.Y. (2022). Phytochemistry, bioactivities and traditional uses of *Michelia* x *alba*. Molecules.

[9] Pushpa V.H., Jayanthi M.K., Rashmi H.R., Shivamurthy V.K.N., Patil S.M., Shirahatti P.S., Ramu R. (2022). New insights on the phytochemical intervention for the treatment of neuropsychiatric disorders using the leaves of *Michelia champaca*: An in vivo and in silico approach. Pharm. Biol..

[10] Babaei G., Aliarab A., Abroon S., Rasmi Y., Aziz S.G. (2018). Application of sesquiterpene lactone: A new promising way for cancer therapy based on anticancer activity. Biomed. Pharmacother..

[11] Shoaib M., Shah I., Ali N., Adhikari A., Tahir M.N., Shah S.W., Ishtiaq S., Khan J., Khan S., Umer M.N. (2017). Sesquiterpene lactone! a promising antioxidant, anticancer and moderate antinociceptive agent from *Artemisia macrocephala* jacquem. BMC Complement. Altern. Med..

[12] Neganova M.E., Afanas’eva S.V., Klochkov S.G., Shevtsova E.F. (2012). Mechanisms of antioxidant effect of natural sesquiterpene lactone and alkaloid derivatives. Bull. Exp. Biol. Med..

[13] Bartikova H., Hanusova V., Skalova L., Ambroz M., Bousova I. (2014). Antioxidant, pro-oxidant and other biological activities of sesquiterpenes. Curr. Top. Med. Chem..

[14] Tang J.Y., Ou-Yang F., Hou M.F., Huang H.W., Wang H.R., Li K.T., Fayyaz S., Shu C.W., Chang H.W. (2019). Oxidative stress-modulating drugs have preferential anticancer effects-involving the regulation of apoptosis, DNA damage, endoplasmic reticulum stress, autophagy, metabolism, and migration. Semin. Cancer Biol..

[15] Bouayed J., Bohn T. (2010). Exogenous antioxidants—Double-edged swords in cellular redox state: Health beneficial effects at physiologic doses versus deleterious effects at high doses. Oxidative Med. Cell. Longev..

[16] Jalal S., Ahmad B., Zhang T., Guo L., Huang L. (2020). SANTAMARINE: Mechanistic studies on multiple diseases. Chem. Biol. Drug Des..

[17] Talapatra S.K., Patra A., Talapatra B. (1973). Parthenolide and a new germacranolide, 11, 13-dehydrolanuginolide, from *Michelia lanuginosa*. Phytochemistry.

[18] Li Y., Ni Z.Y., Zhu M.C., Dong M., Wang S.M., Shi Q.W., Zhang M.L., Wang Y.F., Huo C.H., Kiyota H. (2012). Antitumour activities of sesquiterpene lactones from *Inula helenium* and *Inula japonica*. Z. Für Naturforschung C.

[19] Oh J.H., Kim J., Karadeniz F., Kim H.R., Park S.Y., Seo Y., Kong C.S. (2021). Santamarine shows anti-photoaging properties via inhibition of MAPK/AP-1 and stimulation of TGF-β/Smad signaling in UVA-irradiated HDFs. Molecules.

[20] Zhang J., Xu Q., Yang H.Y., Yang M., Fang J., Gao K. (2021). Inhibition of thioredoxin reductase by santamarine conferring anticancer effect in HeLa cells. Front. Mol. Biosci..

[21] Mehmood T., Maryam A., Tian X., Khan M., Ma T. (2017). Santamarine inhibits NF-kB and STAT3 activation and induces apoptosis in HepG2 liver cancer cells via oxidative stress. J. Cancer.

[22] Wu X., Zhu H., Yan J., Khan M., Yu X. (2017). Santamarine inhibits NF-kappaB activation and induces mitochondrial apoptosis in A549 lung adenocarcinoma cells via oxidative stress. Biomed. Res. Int..

[23] Koc E., Celik-Uzuner S., Uzuner U., Cakmak R. (2018). The detailed comparison of cell death detected by annexin V-PI counterstain using fluorescence microscope, flow cytometry and automated cell counter in mammalian and microalgae cells. J. Fluoresc..

[24] Boice A., Bouchier-Hayes L. (2020). Targeting apoptotic caspases in cancer. Biochim. Biophys. Acta (BBA)-Mol. Cell Res..

[25] Lee C.H., Shih Y.L., Lee M.H., Au M.K., Chen Y.L., Lu H.F., Chung J.G. (2017). Bufalin induces apoptosis of human osteosarcoma U-2 OS cells through endoplasmic reticulum stress, caspase- and mitochondria-dependent signaling pathways. Molecules.

[26] Chou W.H., Liu K.L., Shih Y.L., Chuang Y.Y., Chou J., Lu H.F., Jair H.W., Lee M.Z., Au M.K., Chung J.G. (2018). Ouabain induces apoptotic cell death through caspase- and mitochondria-dependent pathways in human osteosarcoma U-2 OS cells. Anticancer Res..

[27] Kauffman M.E., Kauffman M.K., Traore K., Zhu H., Trush M.A., Jia Z., Li Y.R. (2016). MitoSOX-based flow cytometry for detecting mitochondrial ROS. React. Oxyg. Species.

[28] Shiau J.P., Chuang Y.T., Yang K.H., Chang F.R., Sheu J.H., Hou M.F., Jeng J.H., Tang J.Y., Chang H.W. (2022). Brown algae-derived fucoidan exerts oxidative stress-dependent antiproliferation on oral cancer cells. Antioxidants.

[29] Aldossary S.A. (2019). Review on pharmacology of cisplatin: Clinical use, toxicity and mechanism of resistance of cisplatin. Biomed. Pharmacol. J..

[30] Tang C., Livingston M.J., Safirstein R., Dong Z. (2023). Cisplatin nephrotoxicity: New insights and therapeutic implications. Nat. Rev. Nephrol..

[31] Teppo H.R., Soini Y., Karihtala P. (2017). Reactive oxygen species-mediated mechanisms of action of targeted cancer therapy. Oxidative Med. Cell. Longev..

[32] Wang J., Sun D., Huang L., Wang S., Jin Y. (2021). Targeting reactive oxygen species capacity of tumor cells with repurposed drug as an anticancer therapy. Oxidative Med. Cell. Longev..

[33] Shiau J.P., Chuang Y.T., Tang J.Y., Chen S.R., Hou M.F., Jeng J.H., Cheng Y.B., Chang H.W. (2022). Antiproliferation effects of marine-sponge-derived methanol extract of *Theonella swinhoei* in oral cancer cells in vitro. Antioxidants.

[34] Di Giacomo C., Malfa G.A., Tomasello B., Bianchi S., Acquaviva R. (2023). Natural compounds and glutathione: Beyond mere antioxidants. Antioxidants.

[35] Lu J., Holmgren A. (2014). The thioredoxin antioxidant system. Free Radic. Biol. Med..

[36] Lee H.A., Chu K.B., Moon E.K., Kim S.S., Quan F.S. (2020). Sensitization to oxidative stress and G2/M cell cycle arrest by histone deacetylase inhibition in hepatocellular carcinoma cells. Free Radic. Biol. Med..

[37] Chung T.W., Lin S.C., Su J.H., Chen Y.K., Lin C.C., Chan H.L. (2017). Sinularin induces DNA damage, G2/M phase arrest, and apoptosis in human hepatocellular carcinoma cells. BMC Complement. Altern. Med..

[38] Redza-Dutordoir M., Averill-Bates D.A. (2016). Activation of apoptosis signalling pathways by reactive oxygen species. Biochim. Biophys. Acta.

[39] Xia W., Spector S., Hardy L., Zhao S., Saluk A., Alemane L., Spector N.L. (2000). Tumor selective G2/M cell cycle arrest and apoptosis of epithelial and hematological malignancies by BBL22, a benzazepine. Proc. Natl. Acad. Sci. USA.

[40] Lee E.J., Min H.Y., Joo Park H., Chung H.J., Kim S., Nam Han Y., Lee S.K. (2004). G2/M cell cycle arrest and induction of apoptosis by a stilbenoid, 3,4,5-trimethoxy-4′-bromo-cis-stilbene, in human lung cancer cells. Life Sci..

[41] Zhang R., Loganathan S., Humphreys I., Srivastava S.K. (2006). Benzyl isothiocyanate-induced DNA damage causes G2/M cell cycle arrest and apoptosis in human pancreatic cancer cells. J. Nutr..

[42] Kajstura M., Halicka H.D., Pryjma J., Darzynkiewicz Z. (2007). Discontinuous fragmentation of nuclear DNA during apoptosis revealed by discrete “sub-G1“ peaks on DNA content histograms. Cytom. Part A.

[43] Shiau J.P., Lee M.Y., Tang J.Y., Huang H., Lin Z.Y., Su J.H., Hou M.F., Cheng Y.B., Chang H.W. (2022). Marine sponge *Aaptos suberitoid* extract improves antiproliferation and apoptosis of breast cancer cells without cytotoxicity to normal cells in vitroes. Pharmaceuticals.

[44] Chen B.H., Chang H.W., Huang H.M., Chong I.W., Chen J.S., Chen C.Y., Wang H.M. (2011). (-)-Anonaine induces DNA damage and inhibits growth and migration of human lung carcinoma h1299 cells. J. Agric. Food Chem..

[45] Cheon C., Ko S.G. (2022). Synergistic effects of natural products in combination with anticancer agents in prostate cancer: A scoping review. Front. Pharmacol..

[46] Sauter E.R. (2020). Cancer prevention and treatment using combination therapy with natural compounds. Expert. Rev. Clin. Pharmacol..

[47] Dasari S., Njiki S., Mbemi A., Yedjou C.G., Tchounwou P.B. (2022). Pharmacological effects of cisplatin combination with natural products in cancer chemotherapy. Int. J. Mol. Sci..

[48] Wu J., Li Y., He Q., Yang X. (2023). Exploration of the use of natural compounds in combination with chemotherapy drugs for tumor treatment. Molecules.

[49] Robinson P., Covenas R., Munoz M. (2023). Combination therapy of chemotherapy or radiotherapy and the neurokinin-1 receptor antagonist aprepitant: A new antitumor strategy?. Curr. Med. Chem..

[50] Gonzalez-Moles M.A., Ramos-Garcia P., Esteban F. (2021). Significance of the overexpression of substance P and its receptor NK-1R in head and neck carcinogenesis: A systematic review and meta-analysis. Cancers.

[51] Chow R., Tsao M., Chiu L., Popovic M., Milakovic M., Lam H., DeAngelis C. (2018). Efficacy of the combination neurokinin-1 receptor antagonist, palonosetron, and dexamethasone compared to others for the prophylaxis of chemotherapy-induced nausea and vomiting: A systematic review and meta-analysis of randomized controlled trials. Ann. Palliat. Med..

[52] Garcia-Aranda M., Tellez T., McKenna L., Redondo M. (2022). Neurokinin-1 receptor (NK-1R) antagonists as a new strategy to overcome cancer resistance. Cancers.

[53] Lee H.Y.J., Meng M., Liu Y., Su T., Kwan H.Y. (2021). Medicinal herbs and bioactive compounds overcome the drug resistance to epidermal growth factor receptor inhibitors in non-small cell lung cancer. Oncol. Lett..

[54] Jiao L., Xu J., Sun J., Chen Z., Gong Y., Bi L., Lu Y., Yao J., Zhu W., Hou A. (2019). Chinese herbal medicine combined with EGFR-TKI in EGFR mutation-positive advanced pulmonary adenocarcinoma (CATLA): A multicenter, randomized, double-blind, placebo-controlled trial. Front. Pharmacol..

[55] Nisar S., Masoodi T., Prabhu K.S., Kuttikrishnan S., Zarif L., Khatoon S., Ali S., Uddin S., Akil A.A., Singh M. (2022). Natural products as chemo-radiation therapy sensitizers in cancers. Biomed. Pharmacother..

[56] Park E., Chung S.W. (2019). ROS-mediated autophagy increases intracellular iron levels and ferroptosis by ferritin and transferrin receptor regulation. Cell Death Dis..

[57] Shiau J.P., Chuang Y.T., Tang J.Y., Yang K.H., Chang F.R., Hou M.F., Yen C.Y., Chang H.W. (2022). The impact of oxidative stress and AKT pathway on cancer cell functions and its application to natural products. Antioxidants.

[58] Huang C.H., Yeh J.M., Chan W.H. (2018). Hazardous impacts of silver nanoparticles on mouse oocyte maturation and fertilization and fetal development through induction of apoptotic processes. Environ. Toxicol..

[59] Wang T.S., Lin C.P., Chen Y.P., Chao M.R., Li C.C., Liu K.L. (2018). CYP450-mediated mitochondrial ROS production involved in arecoline N-oxide-induced oxidative damage in liver cell lines. Environ. Toxicol..

[60] Hsieh P.L., Liao Y.W., Hsieh C.W., Chen P.N., Yu C.C. (2020). Soy isoflavone genistein impedes cancer stemness and mesenchymal transition in head and neck cancer through activating miR-34a/RTCB axis. Nutrients.

[61] Chen S.Y., Chang Y.L., Liu S.T., Chen G.S., Lee S.P., Huang S.M. (2021). Differential cytotoxicity mechanisms of copper complexed with disulfiram in oral cancer cells. Int. J. Mol. Sci..

[62] Huang W.Z., Liu T.M., Liu S.T., Chen S.Y., Huang S.M., Chen G.S. (2023). Oxidative status determines the cytotoxicity of ascorbic acid in human oral normal and cancer cells. Int. J. Mol. Sci..

[63] Wong D.Y., Chang K.W., Chen C.F., Chang R.C. (1990). Characterization of two new cell lines derived from oral cavity human squamous cell carcinomas—OC1 and OC2. J. Oral Maxillofac. Surg..

[64] Chen Y.N., Chan Y.H., Shiau J.P., Farooqi A.A., Tang J.Y., Chen K.L., Yen C.Y., Chang H.W. (2024). The neddylation inhibitor MLN4924 inhibits proliferation and triggers apoptosis of oral cancer cells but not for normal cells. Environ. Toxicol..

[65] Lin S.J., Huang C.C. (2022). Strontium peroxide-loaded composite scaffolds capable of generating oxygen and modulating behaviors of osteoblasts and osteoclasts. Int. J. Mol. Sci..

[66] Vignon C., Debeissat C., Georget M.T., Bouscary D., Gyan E., Rosset P., Herault O. (2013). Flow cytometric quantification of all phases of the cell cycle and apoptosis in a two-color fluorescence plot. PLoS ONE.

[67] Fan H.C., Hsieh Y.C., Li L.H., Chang C.C., Janouskova K., Ramani M.V., Subbaraju G.V., Cheng K.T., Chang C.C. (2020). Dehydroxyhispolon methyl ether, a hispolon derivative, inhibits WNT/beta-catenin signaling to elicit human colorectal carcinoma cell apoptosis. Int. J. Mol. Sci..

[68] Liu Y.C., Peng B.R., Hsu K.C., El-Shazly M., Shih S.P., Lin T.E., Kuo F.W., Chou Y.C., Lin H.Y., Lu M.C. (2020). 13-Acetoxysarcocrassolide exhibits cytotoxic activity against oral cancer cells through the interruption of the Keap1/Nrf2/p62/SQSTM1 pathway: The need to move beyond classical concepts. Mar. Drugs.

[69] Sgarbi G., Gorini G., Liuzzi F., Solaini G., Baracca A. (2018). Hypoxia and IF(1) expression promote ROS decrease in cancer cells. Cells.

